# Synergistically enhanced Ni-MOF/CNTs nanocomposite as an electrochemical platform for ultrasensitive determination of sotagliflozin in various matrices, with whiteness and blueness assessments

**DOI:** 10.1007/s00604-025-07235-5

**Published:** 2025-05-26

**Authors:** Hend Z. Yamani, Yasmine H. Hassan, Nancy Magdy, Maha F. Abdel Ghany, Mohammed M. Gomaa

**Affiliations:** 1 Pharmaceutical Analytical Chemistry Department, Faculty of Pharmacy, Ain Shams University, Cairo, 11566 Egypt; 2 Solid State Physics Department, National Research Centre, Giza, 12622 Egypt

**Keywords:** Sotagliflozin, Ni-MOF/CNTs, Nanocomposite, Electrochemical sensor, Whiteness assessment, Blueness assessment

## Abstract

An electrochemical sensing platform based on a nickel metal–organic framework/carbon nanotubes (Ni-MOF/CNTs) nanocomposite has been developed for the determination of sotagliflozin, the first dual sodium-glucose co-transporter inhibitor recently approved by the FDA. The nanocomposite was synthesized using a simple one-pot solvothermal method and characterized by Raman spectroscopy, Fourier-transform infrared spectroscopy (FTIR), X-ray photoelectron spectroscopy (XPS), scanning electron microscope (SEM), and electrochemical impedance spectroscopy (EIS). The Ni-MOF/CNTs nanocomposite demonstrates superior electrochemical properties compared with its monocomponent counterparts, due to the synergistic combination of the advantages of both materials. The electrochemical behavior and the oxidation mechanism of sotagliflozin at the electrode surface were investigated using cyclic voltammetry (CV). Under optimal conditions, the sensor exhibits wide linearity across two concentration ranges: 8.0 × 10^−10^ to 6.0 × 10^−7^ M and 6.0 × 10^−7^ to 8.0 × 10^−5^ M, using differential pulse voltammetry (DPV) with a detection limit of 2.65 × 10^−10^ M. Good recoveries (96.60–102.83%) were achieved in human plasma, urine, and tablets. The whiteness assessment confirms the method’s sustainability, while the blueness assessment demonstrates its applicability. The sensor’s outstanding performance addresses the unmet need for a simple and accurate tool for sotagliflozin determination in biological and pharmaceutical samples, as no existing analytical methods are available for its determination in different matrices. This makes it a valuable tool for sotagliflozin therapeutic drug monitoring, point-of-care diagnostics, and quality control.

**Graphical Abstract:**

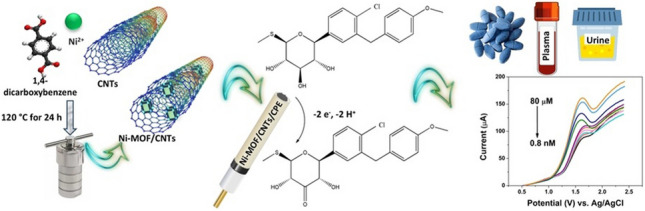

**Supplementary Information:**

The online version contains supplementary material available at 10.1007/s00604-025-07235-5.

## Introduction

Diabetes mellitus (DM) is a rising global health concern, affecting approximately 536.6 million people aged 20–79, or 10.5% of that population. This prevalence is projected to increase to 12.2% by 2045 [1]. DM often leads to long-term complications, placing a significant burden on patients. One prevalent complication is heart failure, affecting about 22% of diabetic patients and is associated with high mortality risk [2]. Fortunately, progress in DM management is accompanied by decreasing rates of these complications [3].

Sotagliflozin, the first dual sodium-glucose co-transporter (SGLT) inhibitor, received FDA approval in May 2023 to lower cardiovascular death risk and heart failure hospitalization in patients suffering from type 2 DM and chronic kidney disease, as well as other cardiovascular risk factors [4]. Effective monitoring of sotagliflozin is essential to optimize dosing, ensure safety and efficacy, and address individual variability, particularly in patients with complex health conditions that sotagliflozin is designed to support. Moreover, the quality control of dosage forms is of great concern to guarantee some criteria such as efficacy, safety, and consistency. Accordingly, there is a critical need for a simple, rapid, sensitive, accurate, and economical analytical tool capable of analyzing sotagliflozin accurately in different biological fluids, and its dosage form.

Compared to other analytical techniques, electrochemical sensors provide several advantages such as short analysis time, high sensitivity, versatility, portability, ability to miniaturize, environmental friendliness, and cost-effectiveness. Furthermore, they require no or minimal sample pretreatment steps. Additionally, they are easy to produce, use, and handle [5, 6]. Carbon paste electrodes (CPE) present several benefits, including simplicity in fabrication, affordability, and easiness in renewing the surface, which prevents electrode fouling. Besides, they exhibit low residual current across a wide potential range. Modifying CPE with different nanomaterials enhances electrocatalytic activity, improves electron transfer, and increases surface area and conductivity [7, 8].

Metal–organic frameworks (MOFs) are a category of nanoporous crystalline networks composed of redox-active metal clusters or ions coordinated to organic ligands [9]. Currently, MOFs have gained growing interest thanks to their impressive features, including microporosity, high specific surface areas, open metal sites, crystalline ordered structure, versatility, and tunability. Due to these unique properties, MOFs have become a hot research topic for applications in the electrochemical field [10].

By selecting different metal ions and organic ligands, MOFs’ characteristics can be precisely controlled. Transition metals like nickel, cobalt, and iron are characterized by hardness, ductility, and high thermal and electrical conductivity. As Ni, Co, and Fe share 3 d^6–8^4 s^2^ valence electron configurations, they possess comparable electrochemical features. Compared to Co, Ni is less toxic and more cost-effective. Despite iron’s abundance, Ni-based materials exhibit better conductivity than those based on iron [11]. Ni-based MOFs serve successfully as electrode modifiers in various electrochemical applications due to their abundance and ability to rapidly form the Ni^2+^/Ni^3+^ redox couple, in addition to superior catalytic activity [12, 13]. Their porous structures, the availability of metal ions for interaction with the electrolyte, and high surface areas improve electron transfer compared to other nickel-based materials like nickel hydroxides/oxides [14]. These properties offer abundant electrocatalytic active sites and transferring pathways, which provide rapid responses to target materials. Despite these unique properties, the poor conductivity, redox activity, and chemical instability of MOFs in aqueous solutions constrain their further application in electrochemical sensors [15]. Moreover, the stacking of pure MOF layers limits the accessibility of metal ion catalytic sites and obstructs electron transfer, resulting in reduced electrochemical performance [16]. To address these drawbacks and promote the involvement of metal centers in fast electron transfer processes, materials with high conductivity, especially carbon-based ones, have been incorporated with MOFs to create composites that show enhanced electrochemical activity, conductivity, and detection sensitivity, paving the way for MOF-based hybrid materials in electrochemistry. Numerous MOF-based materials, such as Cu-MOF [17] and Zr-MOF [18], when modified with carbon-based materials, have revealed a synergistic enhancement in the sensing of multiple analytes. CNTs, with their remarkable superior conductivity, high surface area, mechanical strength, and excellent stability [19], are particularly gaining much consideration for combination with MOFs when designing electrochemical sensors [20, 21].

To the best of our knowledge, no fully validated analytical methods have been reported for the determination of sotagliflozin. Therefore, this study aims to develop a highly sensitive voltammetric sensor for assaying sotagliflozin in various matrices based on nickel metal–organic framework/carbon nanotubes (Ni-MOF/CNTs) composite. The proposed approach proves that Ni-MOF/CNTs-modified CPE (Ni-MOF/CNTs/MCPE) exhibits synergistic electrocatalytic activity toward the oxidation of sotagliflozin with high sensitivity, satisfying accuracy results, and reliable stability. The porous framework of Ni-MOF facilitates close contact between sotagliflozin and active sites offering abundant transferring pathways. Additionally, CNTs with their incredible properties improve conductivity and stability. The electrochemical oxidation mechanism of sotagliflozin at the proposed electrode surface was investigated. The promising Ni-MOF/CNTs/MCPE was successfully applied to quantify sotagliflozin in tablets, human plasma, and urine. In addition to being eco-friendly, deliverable, inexpensive, and easy to operate, these pros make it an outstanding choice for routine practice and point-of-care diagnostics.

## Materials and methods

### Materials and chemicals

Sotagliflozin standard was procured from Baoji Guokang Bio-Technology (China). Inpefa® tablets 200 mg were obtained from Lexicon Pharmaceuticals, Inc. (USA). Nickel chloride hexahydrate (NiCl_2_·6H_2_O) and 1,4-dicarboxybenzene were obtained from Sisco Research Laboratories Pvt. Ltd. (India). Multiwalled CNTs, paraffin oil, graphite powder, and ethanol were obtained from Merck (Germany). Sodium hydroxide, N, N-dimethyl-formamide (DMF), o-phosphoric acid, boric acid, glacial acetic acid, sulfuric acid, and nitric acid were acquired from ADWIC (Egypt). De-ionized water was employed for all experiments.

### Instruments

Electrochemical measurements, including CV and DPV, were carried out using a Metrohm AG 884 Professional VA voltammeter (Switzerland) operated with Viva software version 2.1. The setup employed a three-electrode system with an Ag/AgCl electrode (3.00 M KCl) as the reference, a platinum wire as the counter electrode, and CPE serving as the working electrode. EIS measurements were carried out using a Metrohm Autolab PGSTAT204 potentiostat/galvanostat (Netherlands), employing the same three-electrode cell configuration described above.

A Witec Alpha 300 RA confocal Raman microscope (Germany) equipped with a 532-nm laser was utilized to investigate the molecular structure. Functional group characterization was performed using a Vertex 80 V FTIR spectrometer (Germany). The nanocomposite’s chemical composition was analyzed using a Thermo Fisher Scientific K-Alpha XPS (USA). The surface morphology was examined using a Quanta FEG 250 SEM (FEI, USA). A Jenway digital pH meter (model 3510, UK) with a combined glass electrode (924,005) was used to adjust the pH.

### Standard solutions

A standard stock solution of sotagliflozin (1 × 10^−3^ M) was prepared by accurately weighing and dissolving the pure sotagliflozin in ethanol. Following that, the standard working solutions were made by diluting the stock solution appropriately with Britton-Robinson buffer (BRB) of pH 10.0.

### Procedures

#### Synthesis of oxidized MWCNTs

To 80 mL of a mixture of concentrated sulfuric acid and nitric acid (3:1), a weight of 0.25 g of MWCNTs was added cautiously. The temperature was kept at 80 °C for 4 h. The black solid product was repeatedly rinsed with deionized water, then dried at 100 °C overnight.

#### Synthesis of Ni-MOF/CNTs and Ni-MOF

The nanocomposite of Ni-MOF/CNTs was synthesized following the method reported by Wen et al. [22]. Initially, 0.173 g of NiCl_2_·6H_2_O and 0.332 g of 1,4-dicarboxybenzene were dissolved in 10 mL of DMF (solution A). Based on the total mass of nickel salts, 5 wt% of the oxidized MWCNTs were dispersed in 20 mL of DMF using ultrasonication for 30 min (suspension B). Solution A was then added to suspension B, and the mixture was sonicated for an additional 30 min. To a Teflon-lined stainless-steel autoclave with 70 mL capacity, this mixture was subsequently transferred, sealed, and heated at 120 °C for 24 h. The green solid product was filtered, repeatedly washed with ethanol, and dried in air, as presented in Fig. S1. The Ni-MOF was synthesized following the same procedure, with the exception that CNTs were not added to the 20 mL of DMF.

#### Fabrication of working electrodes

Graphite powder (0.30 g) was thoroughly mixed with paraffin oil (0.18 mL) until a homogeneous paste was formed for the preparation of the bare CPE. The hole of the electrode was firmly filled with the prepared carbon paste. The electrode surface was then smoothed by polishing on filter paper.

The modified electrode (Ni-MOF/CNTs/MCPE) was prepared by incorporating 7% w/w of Ni-MOF/CNTs into the carbon paste, which was then filled into the electrode body in addition to being polished in the same manner as bare CPE.

#### Electrochemical measurements

To optimize the modifier type and its (w/w)% ratio, select the appropriate supporting electrolyte, and investigate the electrochemical behavior and oxidation mechanism of sotagliflozin at the electrode surface, CV was performed using 1 × 10^−4^ M sotagliflozin in BRB (pH 10.0) over a potential range of − 0.5 to + 2.0 V vs Ag/AgCl at a scan rate of 100 mV s^−1^, unless otherwise specified.

For studying the electrodes electroactive surface area, and reaction transfer kinetics, CV and EIS measurements were performed in 1 × 10^−3^ M K_3_Fe(CN)_6_^3−/4−^ in 0.1 M KCl solution over a frequency range from 100 kHz to 0.01 Hz.

For the quantitative determination of sotagliflozin, DPV was applied with a potential window 0.0–2.4 V. The optimal conditions for measurements are as follows: a step potential of 6.0 mV, a potential step time of 0.10 s, a scan rate of 60 mV s^−1^, a pulse amplitude of 64 mV, and a pulse time of 0.06 s. Calibration plots were constructed by plotting peak current (*I*_p_) of sotagliflozin at 1.65 V vs Ag/AgCl against the corresponding concentration in the range of 8.0 × 10^−10^–8.0 × 10^−5^ M prepared in BRB at pH 10.0.

The working electrode surface was carefully polished before each measurement to ensure a reproducible surface.

#### Application in pharmaceutical formulation

Ten Inpefa® tablets were finely ground, and an amount equivalent to 200 mg of sotagliflozin was accurately weighed and transferred into a 100-mL volumetric flask, then diluted to volume with ethanol. After 10 min of sonication then filtration, an 85-µL aliquot was further diluted with BRB (pH 10.0) in a 10-mL flask. The solution was analyzed using DPV under the specified conditions in the “Electrochemical measurements” section. The average recovery% and %RSD were calculated. Standard addition technique was employed to validate the proposed method.

#### Application in human plasma and urine samples

Human plasma was sourced from VACSERA, the Egyptian Holding Company for Biological Products and Vaccines (Egypt), and fresh urine was sampled from healthy individuals (under the approval of the Research Ethics Committee at Faculty of Pharmacy, Ain Shams University, Cairo, Egypt). Clinical trial number: not applicable.

For plasma samples, 20 µL of sotagliflozin standard solutions was spiked into 480 µL of human plasma and vortexed. Then, 1.5 mL of methanol was added for protein precipitation. After centrifugation (3000 rpm, 15 min), the supernatant was evaporated, reconstituted with BRB (pH 10.0), and diluted to volume in a set of 10-mL volumetric flasks.

Urine samples of 10 µL were added to a series of 10-mL volumetric flasks and spiked with varying concentrations of sotagliflozin. The mixtures were diluted with BRB at pH 10.0 to reach the final volume, without any additional pretreatment.

The prepared plasma and urine samples were analyzed using DPV under the conditions detailed in the “Electrochemical measurements” section. The average recovery% and %RSD were calculated.

## Results and discussion

### Characterization of Ni-MOF/CNTs nanocomposite

**Fig. 1:**
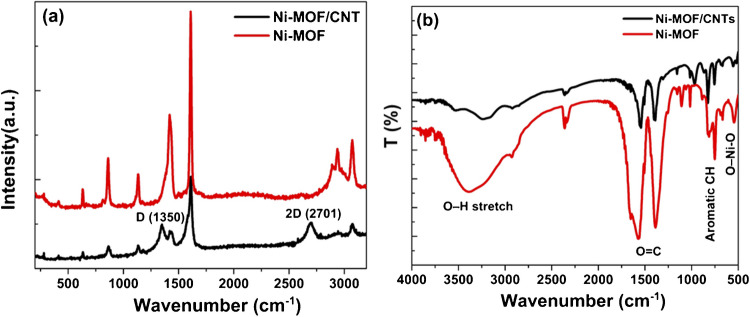
**a** Raman spectra, and **b** FTIR spectra of Ni-MOF and Ni-MOF/CNTs nanocomposite

Raman spectroscopy was employed to examine the chemical structure of Ni-MOF, confirming its integration with CNTs. As shown in Fig. 1a, the Raman spectra reveal the D and G bands of Ni-MOF at 1421 cm^−1^ and 1610 cm^−1^, respectively, in agreement with previously reported values for MOF structures [22, 23]. The D band of CNTs is observed at 1350 cm^−1^, while their G band overlaps with that of Ni-MOF. Additionally, the prominent D and 2D peaks at 1350 cm^−1^ and 2701 cm^−1^, respectively, provide strong evidence for the successful combination between Ni-MOF and CNTs.

Fourier-transform infrared spectroscopy (FTIR) analysis is an effective technique to identify the functional groups present in materials. The FTIR spectrum (Fig. 1b) reveals that both Ni-MOF and Ni-MOF/CNT include vibration modes corresponding to -COO-, OH^−^, CH groups, and O-Ni–O bonds. The Ni-MOF sample has a significant peak at 3396 cm^−1^, which is due to the OH group. The aromatic ring is represented by peaks at 1570, 1387, 826, and 753 cm^−1^. The peaks at 826 and 753 cm^−1^ correspond to para-aromatic CH stretching mode. Furthermore, the two significant bands at 1570 and 1387 cm^−1^ correspond to the coordinated carboxyl group’s asymmetric and symmetric stretching vibrations. A peak at 535 cm^−1^ indicates the O-Ni–O bond, which aligns with previous research results [24]. These results indicate the effective synthesis of Ni-MOF. Similarly, the Ni-MOF/CNTs composite demonstrates the functional groups of Ni-MOF with decreasing the intensity of the hydroxyl group. In addition, a slight peak shift was noticed in the vibrations of -COO-, OH-, aromatic CH groups, and O-Ni–O due to CNT interaction with Ni-MOF, as examined by Raman studies.

**Fig. 2:**
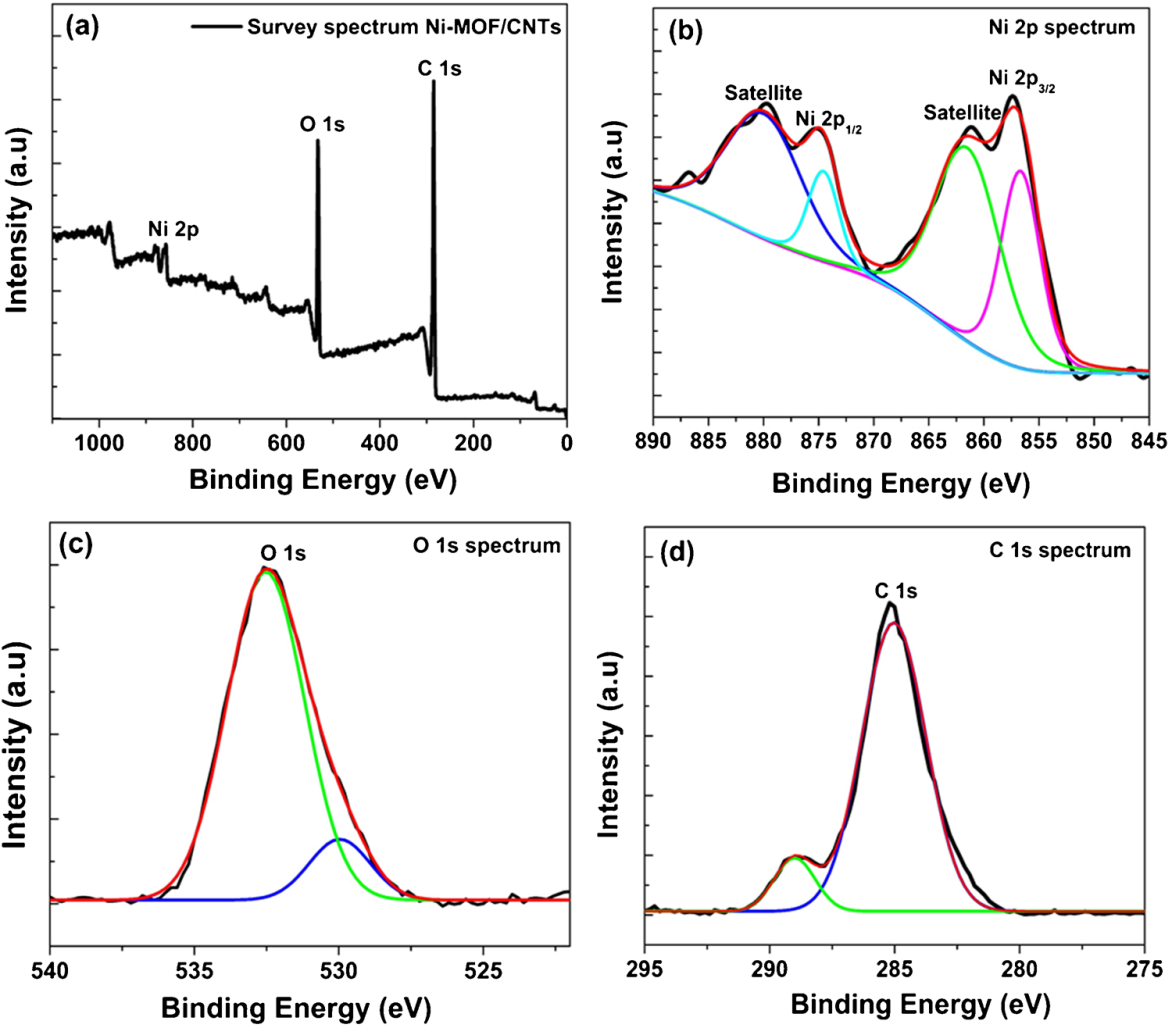
XPS spectra of Ni-MOF/CNTs nanocomposite: **a** the survey spectrum, **b** Ni 2p spectrum, **c** O 1 s spectrum, and **d** C 1 s spectrum

To better understand the chemical composition of the Ni-MOF/CNTs sample, X-ray photoelectron spectroscopy (XPS) characterization was conducted. The results are shown in Fig. 2. The survey spectrum (Fig. 2a) reveals signals exclusively from C, O, and Ni elements.

The high-resolution Ni 2p spectrum (Fig. 2b) demonstrates that the Ni 2p_3_/_2_ and Ni 2p₁/_2_ peaks appear at binding energies of 856.72 eV and 874.63 eV, respectively, with additional satellite features observed at 861.56 eV and 880 eV. This suggests the presence of nickel in the divalent state. Furthermore, the high-resolution O 1 s spectrum (Fig. 2c) displays two peaks at 530 eV and 532.52 eV binding energies, which are attributed to hydroxyl groups and chemisorbed water, respectively [25]. In addition, the fitted XPS spectrum of C 1 s (Fig. 2d) shows two peaks at 285.03 eV and 289.99 eV, which belong to the C–C and O-C = O states, accordingly [26].

**Fig. 3:**
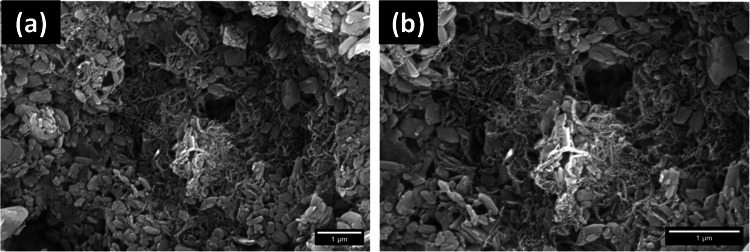
SEM images of Ni-MOF/CNTs nanocomposite at **a** lower magnification and **b** higher magnification

The surface morphology of the Ni-MOF/CNTs nanocomposite was examined using scanning electron microscope (SEM), as shown in Fig. 3a and b. The SEM images reveal a porous structure characterized by a network of entangled CNTs strands that are uniformly embedded and interwoven throughout a framework of flake-like Ni-MOF crystallites, forming a scaffold that enhances the composite’s surface area and structural integrity. This scaffold acted as a nucleus for the growth and assembly of Ni-MOF crystals while also reducing the clustering of Ni-MOF flakes. The SEM micrographs indicate that the nanocomposite is characterized by penetrable pores that are expected to facilitate electron transfer and promote the accessibility of electroactive sites. The observed morphology confirms the successful integration of both components, supporting their synergistic role in enhancing the electrochemical performance of the modified electrode.

### Electrochemical behavior of sotagliflozin

**Fig. 4:**
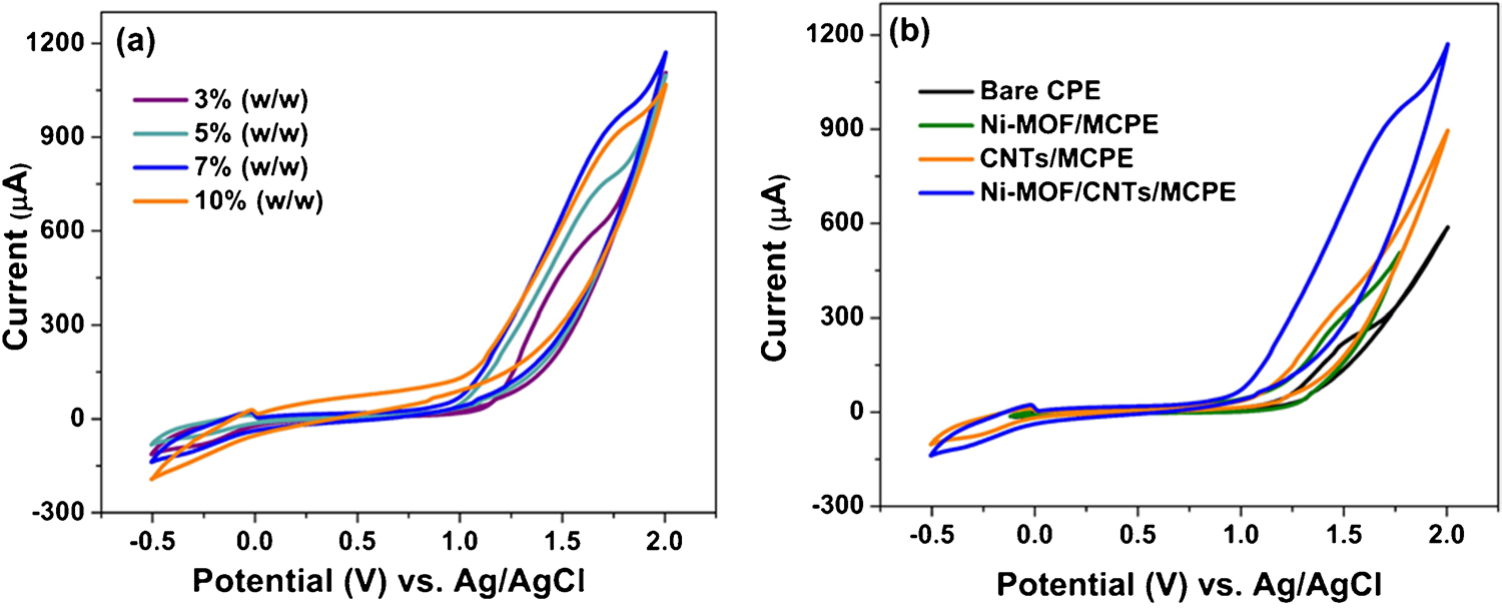
CV responses of **a** different % (w/w) of Ni-MOF/CNTs/MCPEs, and **b** bare CPE, Ni-MOF/MCPE, CNTs/MCPE, and 7% (w/w) Ni-MOF/CNTs/MCPE, in 1 × 10^−4^ M of sotagliflozin in BRB of pH 10 at a scan rate of 100 mV s^−^.^1^

For the investigation of its electrochemical behavior, sotagliflozin was subjected to a CV scan. The CV of 1 × 10^−4^ M sotagliflozin in BRB (pH 10.0) was recorded in the potential range from − 0.5 to + 2.0 V at a scan rate (*ν*) = 100 mV s^−1^. Sotagliflozin yields a single definite anodic peak at + 1.65 V using Ni-MOF/CNTs/MCPE (Fig. 4b). There was no cathodic peak on the reverse scan, indicating the irreversible oxidation nature of sotagliflozin.

### Experimental conditions optimization

#### Effect of the modifier and its concentration

The effect of various concentrations of Ni-MOF/CNTs on the *I*_p_ of 1 × 10^−4^ M sotagliflozin was investigated in BRB (pH 10.0) applying CV at a scan rate of 100 mV s^−1^. Varying the concentrations of Ni-MOF/CNTs from 3.0 to 10.0% (w/w) revealed that 7.0% (w/w) Ni-MOF/CNTs yielded the highest electrochemical response (Fig. 4a), indicating it as the optimal composition.

It is clearly seen in Fig. 4b that modifying the CPE notably enhances the *I*_p_. The bare CPE showed poor response where the oxidation peak cannot be well detected. Using Ni-MOF or CNTs resulted in *I*_p_ enhancement. However, the combination of CNTs and Ni-MOF in a nanocomposite produces the maximum *I*_p_.

CV responses were recorded at various scan rates using 1.0 mM Fe(CN)_6_^4−/3−^ in 0.1 M KCl to assess the electrochemically active surface areas of both bare and modified electrodes (Fig. S2). According to Randles–Sevcik equation: *I*_p_ = 2.65 × 10^5^*n*^3/2^*AD*^1/2^*Cv*^1/2^, where *I*_p_ represents the peak current, *n* is the number of electrons involved in the electrochemical process, *A* is the electroactive surface area of the sensor (cm^2^), *D* is the diffusion coefficient (cm^2^ s^−1^), *C* is Fe(CN)_6_^4−/3−^ concentration (mol cm^−3^), and *ν* is the employed scan rate (V s^−1^). For Fe(CN)_6_^4−/3−^, *n* equals 1, while *D* equals 7.6 × 10^−6^ cm^2^ s^−1^. By using the slope of *I*_p_ against the *v*^1/2^ plot, the surface areas are calculated. The bare CPE exhibits the lowest surface area (0.027 cm^2^). Incorporation of Ni-MOF leads to a modest increase (0.034 cm^2^) due to its intrinsic porosity, which provides additional exposed sites for electrochemical interaction. A greater enhancement was achieved with CNTs/MCPE (0.055 cm^2^), as CNTs offer a high surface-to-volume ratio and a network-like architecture that increases the accessible electrode surface. The Ni-MOF/CNTs composite exhibits the highest surface area (0.095 cm^2^), benefiting from the combination of MOF porosity and the high surface area of CNTs, which synergistically enhances the available surface area for electrochemical activity. This enhancement is reflected in the *I*_p_ of sotagliflozin in BRB (pH 10.0) (Fig. 4b), where the current increases in the same order, with the nanocomposite (Ni-MOF/CNTs) showing the highest *I*_p_ response.

The electron transfer capability of the electrode plays a vital role in determining the overall performance. EIS measurements were performed using 1.0 mM Fe(CN)_6_^4−/3−^ in 0.1 M KCl. The corresponding Nyquist plots for the bare CPE and MCPEs are presented in Fig. S3. The semicircle diameter in the high-frequency range reflects the charge transfer resistance (*R*_ct_) at the electrode interface. The electron transfer rate constant (*k*^0^) and the exchange current density (*j*_0_) were calculated using the following equations [27, 28]:

$$k^0=\frac{RT}{n^2F^2R_{ct}AC}$$

$$j_0=\frac{RT}{nFR_{ct}}$$

where *R* denotes gas constant (8.314 J K^−1^ mol^−1^), *T* represents the absolute temperature (298 K), and *F* is the Faraday constant (96,485 J). *n*,* A*, and *C* have been defined above. The calculated *R*_ct_,* k*^0^, and* j*_0_ values are listed in Table S1. From the results obtained, the bare CPE exhibited the highest *R*_ct_, and the lowest *k*^0^ and* j*_0_ indicating sluggish electron transfer kinetics and weak catalytic efficiency. Modification with CNTs lowers *R*_ct_ and improves both *k*^0^ and* j*_0_ attributed to the excellent electrical conductivity and high surface area of CNTs. In contrast, the Ni-MOF/MCPE exhibit a higher *R*_ct_, reflecting the intrinsically poor electrical conductivity of the Ni-MOF. Compared to Ni-MOF alone, the incorporation of CNTs into the nanocomposite significantly reduces *R*_ct_ and increases both *k*^0^ and *j*_0_, reflecting enhanced electron transfer kinetics and improved catalytic efficiency [20, 21, 29]. These findings are consistent with the peak-to-peak separation (Δ*E*_p_) values obtained from the CV measurements using the [Fe(CN)_6_]^4−^/^3−^ redox couple (Fig. S2), where Δ*E*_p_ was 288 mV, 244 mV, 182 mV, and 200 mV for the bare CPE, Ni-MOF/MCPE, CNTs/MCPE, and Ni-MOF/CNTs/MCPE, respectively, where a smaller Δ*E*_p_ reflects faster electron transfer kinetics.

MOFs were selected due to their redox activity, and intrinsic porosity, that provides additional exposed sites for electrochemical interaction and facilities electron transfer pathways [10]. Despite the advantages, MOFs face challenges in electrochemical sensors due to poor conductivity, and chemical instability in aqueous solutions, as well as restricted electron transfer from stacked layers [15, 16]. To address these issues, CNTs, known for their excellent conductivity, surface area, and stability, were combined with Ni-MOF to form a composite. The integration of Ni-MOFs and CNTs combines the advantages of both materials. This synergy significantly enhances the electrochemical properties of the Ni-MOF/CNTs/MCPE, improving its electroactive surface area, reducing charge transfer resistance, and enhancing electron transfer kinetics and catalytic activity. As a result, the composite boosts the anodic current response for sotagliflozin oxidation.

#### Effect of the supporting electrolyte and pH

**Fig. 5:**
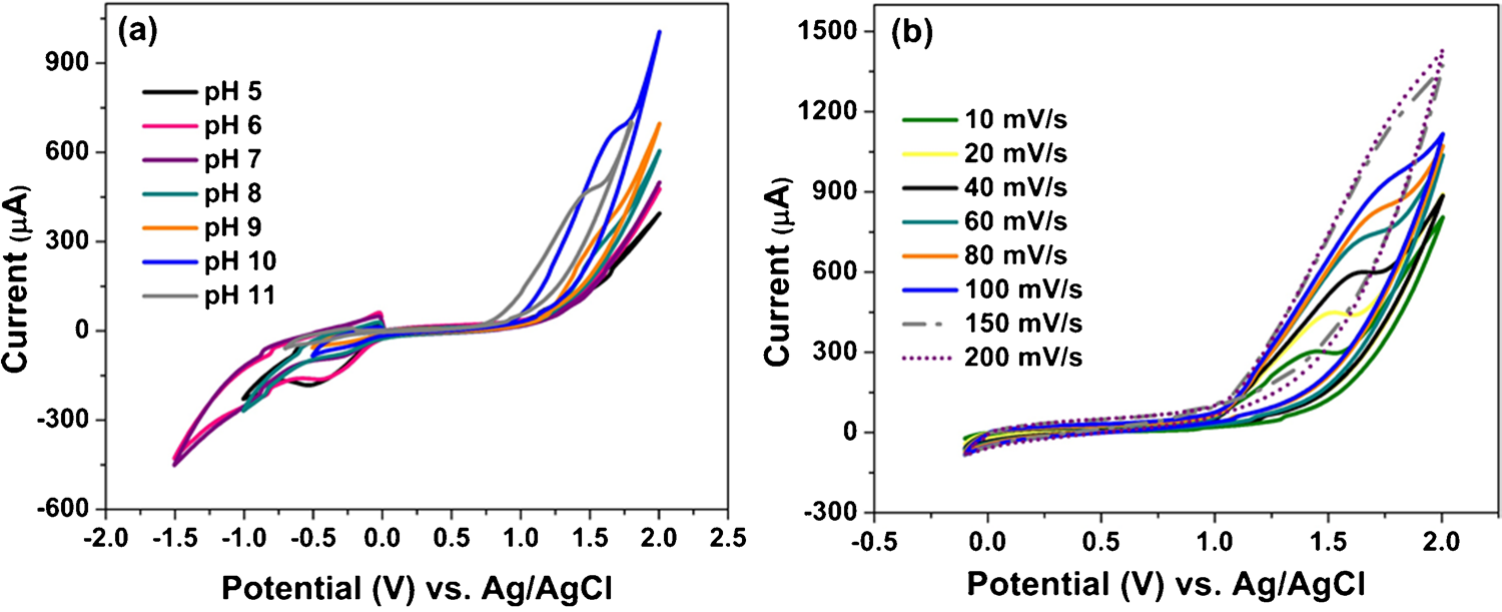
CV responses of Ni-MOF/CNTs/MCPE in 1 × 10^−4^ M of sotagliflozin in BRB **a** over a pH range (5.0–11.0) at a scan rate of 100 mV s^−1^ and **b** at different scan rates (10–200 mV s^−1^) in pH 10.0

To identify the optimal electrolyte for the electrochemical analysis of sotagliflozin, various supporting electrolytes were evaluated by recording CV responses of 1 × 10^−4^ M sotagliflozin using Ni-MOF/CNTs/MCPE at a scan rate of 100 mV s^−1^. Initially, 0.1 M KCl and 0.1 M H_2_SO_4_ were tested; however, no oxidation peak for sotagliflozin was observed. BRB was subsequently investigated due to its broad buffering capacity across a wide pH range, allowing for systematic exploration of pH-dependent electrochemical behavior. An increase in pH leads to a noticeable enhancement in the anodic *I*_p_ and improved peak shape, with the most favorable response observed at pH 10.0 (Fig. 5a). Additional buffers that cover pH 10.0, such as borate buffer and sodium bicarbonate buffer, were also examined. Among all tested systems, BRB yielded the best electrochemical response. Consequently, BRB buffer at pH 10.0 was selected for all subsequent experiments.

The peak potential (*E*_p_) shifts toward lower positive values as the pH increases. This suggests that sotagliflozin oxidation is dependent on pH. A linear relationship between *E*_p_ and the pH is found and can be described by the following linear regression equation: *E*_p_(*V*) = 2.121 − 0.0505 pH (*r*) = 0.9785.

The slope is found to be − 50.5 mV/pH units for Ni-MOF/CNTs/MCPE; this aligns with the Nernstian value of − 59.0 mV, suggesting that the electrochemical oxidation process involves an equal transfer of protons and electrons [29].

#### Effect of scan rate (*ν*)

The influence of various scan rates (*ν*) on *I*_p_ of 1 × 10^−4^ M sotagliflozin was examined by CV in BRB (pH 10.0) with Ni-MOF/CNTs/MCPE. It is evident as the scan rate increases from 10 to 200 mV s^−1^, there is a corresponding increase in the anodic *I*_p_ (Fig. 5b).

The relationship between the *I*_p_ and the square root of the scan rate (*ν*^1/2^) is linear (*r* = 0.9910) and can be represented by the following equation: *I*_p_ = 6.3417 *ν*^1/2^ − 1.8735. This indicates that the diffusion process controls sotagliflozin oxidation at the proposed sensor (Fig. S4). For further confirmation, a linear response is found upon plotting log *I*_p_ versus log *ν* which can be demonstrated by log *I*_p_ = 0.5482 log *ν* + 0.7023 (*r* = 0.9924) (Fig. S5). The slope value is 0.5482, which is close to the ideal slope of 0.5. This further validates that the oxidation mechanism of sotagliflozin is governed by diffusion.

Correspondingly, it is remarked that the oxidation *E*_p_ is dependent on log *ν* (Fig. S6). Consistent with Laviron theory [30], the relationship between *E*_*p*_ and log of scan rate is defined by the following equation: *E*_*p*_ = *E*_*o*_ + (2.303*RT*/*αnF*)log(*RTk*^*0*^/*αnF*) + (2.303*RT*/*αnF*)log* ν.*

where *E*_o_ is the formal potential and *α* is the charge transfer coefficient. From the slope of this equation, the value of *αn* can be found. Typically, *α* is supposed to be 0.5 for irreversible reactions. Accordingly, the number of electrons involved in the oxidation mechanism (*n*) = 2.3 ≈ 2.

### Mechanism of sotagliflozin oxidation

**Fig. 6:**
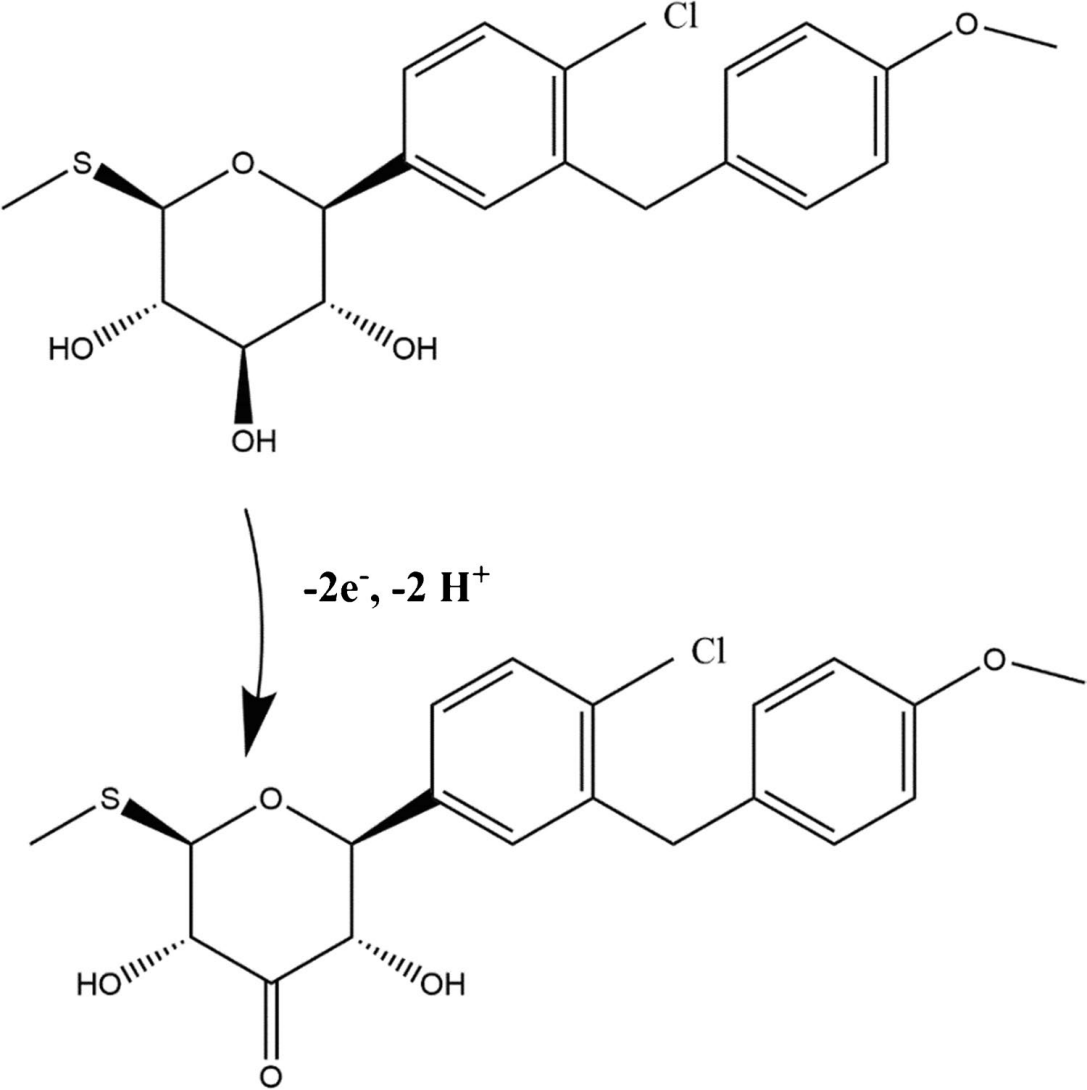
The proposed mechanism of sotagliflozin oxidation at Ni-MOF/CNTs/MCPE

Based on the findings discussed above, obtained by studying the electrochemical behavior of sotagliflozin at various pH levels and scan rates, it can be concluded that the oxidation of sotagliflozin is an irreversible, diffusion-controlled process involving the transfer of two electrons and two protons. The hydroxyl group at the C3 position of the tetrahydropyran ring is the most susceptible to oxidation. As illustrated in Fig. 6, this hydroxyl group undergoes oxidation to form a carbonyl group, resulting in the formation of a ketone. As demonstrated by Wan et al. [31]. through density functional theory studies, Gibbs free energy profiles for the oxidation of hydroxyl groups at the C2, C3, and C4 positions of glucopyranosides indicate that oxidation at C3 is intrinsically favored in the presence of the ring oxygen. β-hydride elimination at C3 exhibits both the lowest activation energy and the most exergonic behavior compared to the corresponding processes at C2 and C4. The inductive effect of the ring oxygen induces positive polarization at the adjacent C1 and C5 positions, making the accumulation of positive charge at neighboring carbons (C2 and C4) energetically unfavorable. In contrast, C3 becomes the most favorable site for β-hydride elimination due to minimized electrostatic repulsion between the positively polarized α-carbon adjacent to the ring oxygen and the carbonyl carbon forming during oxidation. This was further supported by the study of the electrochemical oxidation of glycosides conducted by Kidonakis et al. [32]. Furthermore, C3 bears the most acidic proton, as it is flanked by two hydroxyl groups that exert additional inductive effects, further increasing its susceptibility to oxidation through proton loss.

### Method validation

The proposed voltammetric method was validated per the International Conference on Harmonization (ICH) guidelines. The method validation criteria are presented in Table S2.

**Fig. 7:**
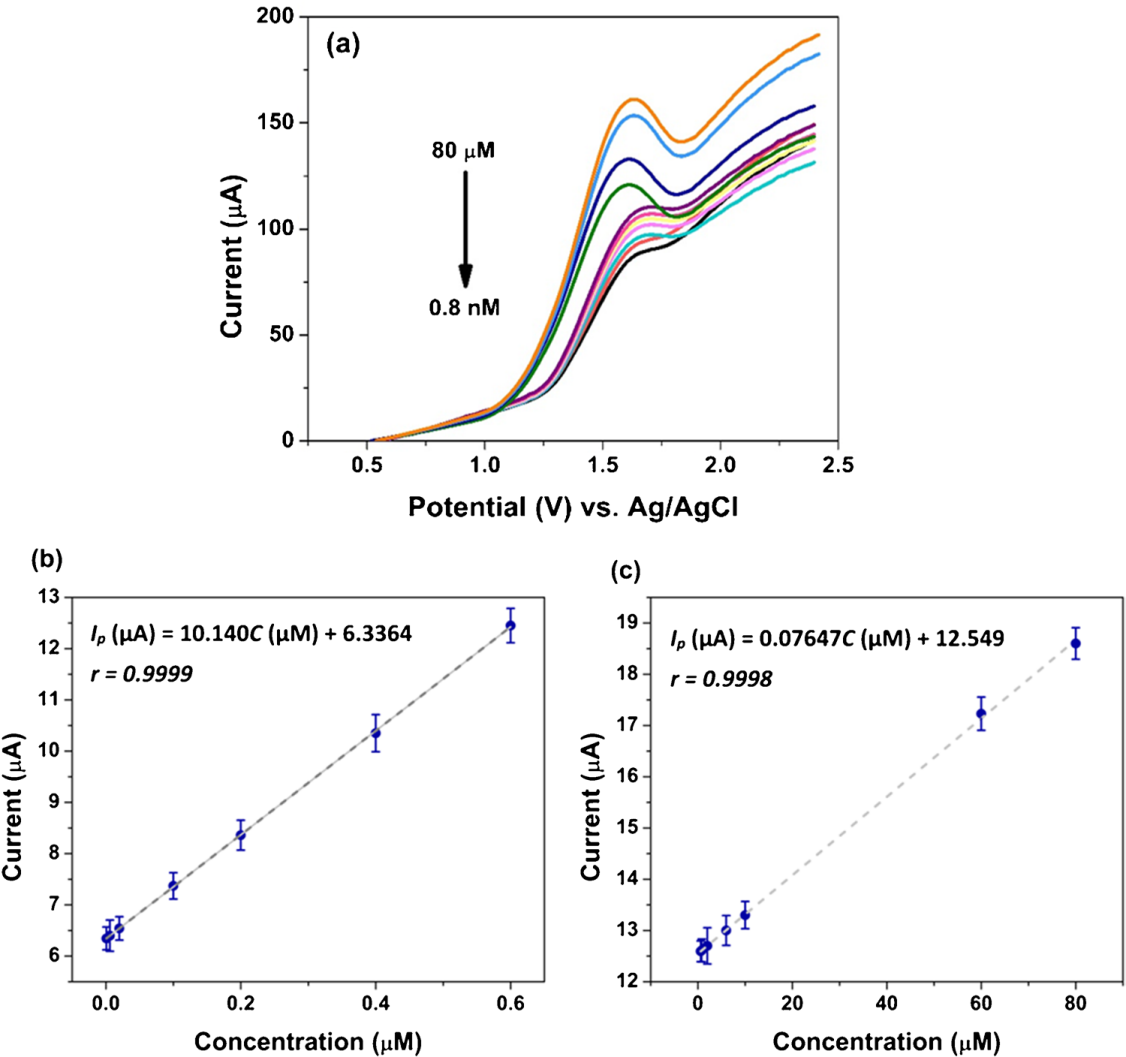
**a** DPV responses of different sotagliflozin concentrations (8.0 × 10^−10^–6.0 × 10^−7^) and (6.0 × 10^−7^–8.0 × 10^−5^) in BRB at pH 10.0 using Ni-MOF/CNTs/MCPE; the calibration plots of sotagliflozin over two linear ranges; **b** (8.0 × 10^−10^–6.0 × 10^−7^ M), and **c** (6.0 × 10^−7^–8.0 × 10^−5^ M)

For the quantitative determination of sotagliflozin, DPV was applied by recording the responses from sotagliflozin solutions at varying concentrations, prepared in BRB at pH 10.0 (Fig. 7a). The response is linear across two segments, covering the concentration ranges of (8.0 × 10^−10^–6.0 × 10^−7^ M) and (6.0 × 10^−7^–8.0 × 10^−5^ M) (Fig. 7b and c). The corresponding regression equations are as follows: *I*_p_ (µA) = 10.140*C* (µM) + 6.3364 (*r* = 0.9999) and *I*_p_ (µA) = 0.07647*C* (µM) + 12.549 (*r* = 0.9998). The limit of detection (LOD) was determined using the formula (3.3 *σ*/*S*); in comparison, the limit of quantification (LOQ) was calculated as (10 *σ*/*S*), where *σ* stands for the standard deviation of response, and *S* represents the slope of the calibration plot. The LOD and LOQ are 2.65 × 10^−10^ M and 7.95 × 10^−10^ M, respectively, revealing that Ni-MOF/CNTs/MCPE is highly sensitive.

The mean percentage recoveries for sotagliflozin in pure form were calculated to confirm the method’s accuracy. It is found to be 99.78% ± 1.438 in the lower linear segment and 98.53% ± 1.270 in the higher one, manifesting the accuracy of the offered sensor. Repeatability (Fig. S7a, b) and intermediate precision were expressed as %RSD, which is found to be < 2%, as presented in Table S2. Regarding reproducibility, three independent Ni-MOF/CNTs/MCPEs were fabricated, and their responses were measured in 1 × 10^−6^ M sotagliflozin solution in BRB of pH 10.0. The %RSD of *I*_p_ is 1.94%, showing adequate reproducibility (Fig. S8a). To test the stability of Ni-MOF/CNTs/MCPE over 60 days, 1 × 10^−5^ M sotagliflozin in BRB at pH 10.0 was used. The response remained stable throughout this period, indicating consistent stability (Fig. S8b).

### Selectivity study

To assess the selectivity of the proposed Ni-MOF/CNTs/MCPE, the effect of various potentially interfering species was investigated with a constant concentration of 1 × 10^−5^ M of sotagliflozin in BRB of pH 10.0 at concentration ratio 1:10 (analyte:interferent). The interfering species examined were regularly used excipients in dosage forms like starch, polyethylene glycol (PEG), microcrystalline cellulose (MCC), magnesium stearate, talc, or biological fluid components as creatinine, uric acid, urea, k^+^, Na^+^, Mg^2+^, bicarbonate, ascorbic acid, and amino acids as leucine, threonine. Moreover, the effects of structurally related and some co-administered drugs were examined in a ratio of 1:1. No significant change in the oxidation peak of sotagliflozin is observed (Fig. S9. and Table S3.). It can be concluded that the suggested sensor demonstrates suitable selectivity for sotagliflozin, effectively distinguishing it even in the presence of interferents typically found in pharmaceutical preparations or biological samples.

### Applications of Ni-MOF/CNTs/MCPE

To examine the adequacy of the developed method, the proposed Ni-MOF/CNTs/MCPE was successfully applied for quantifying sotagliflozin in tablets, plasma, and urine with excellent recoveries, as shown in Table 1. The proposed sensor offers adequate performance with satisfactory recoveries, establishing it as a reliable, promising tool for the assay of sotagliflozin in quality control labs and point-of-care diagnostics.

**Table 1:** Determination of sotagliflozin in Inpefa® tablets, human plasma, and urine using Ni-MOF/CNTs/MCPE

	Claimed concentration (mg)	Found concentration (mg)^*^	Recovery%
	200 mg/tablet	199 mg/tablet	99.5 ± 1.450
	Added concentration (M)	Found concentration (M)^*^	Recovery%
Tablets	1.00 × 10^−8^	1.016 × 10^−8^	101.60
	1.00 × 10^−7^	9.99 × 10^−8^	99.90
	1.00 × 10^−6^	9.94 × 10^−7^	99.40
	**Mean ± %RSD**		100.28 ± 1.164
Plasma	7.00 × 10^−9^	6.98 × 10^−9^	99.71
	1.00 × 10^−8^	9.66 × 10^−9^	96.60
	2.00 × 10^−8^	1.98 × 10^−8^	99.00
	**Mean ± %RSD**		98.44 ± 1.660
Urine	8.00 × 10^−9^	8.03 × 10^−9^	100.38
	8.00 × 10^−8^	7.97 × 10^−8^	99.63
	6.00 × 10^−7^	6.17 × 10^−7^	102.83
	**Mean ± %RSD**		100.95 ± 1.663

^*^Average of three determinations for each concentration

### Whiteness assessment

White analytical chemistry (WAC) extends the green analytical chemistry (GAC) principles by proposing 12 WAC principles serving as an alternative to the 12 GAC principles. Instead of focusing on greenness at the cost of functionality, WAC aligns more closely with sustainability by pursuing a balance between both functional and environmental aspects. Consequently, WAC introduced additional criteria that influence the method’s quality. The RGB12 algorithm is used to assess these criteria, splitting them into three categories: red, green, and blue which collectively produce the appearance of whiteness. Each group consists of four algorithms. The red group (R1–R4) focuses on validation parameters: scope of application, LOD with LOQ, precision, and accuracy. The green group (G1–G4) evaluates aspects of green analytical chemistry, namely, reagents toxicity, waste, energy consumption, and direct impacts on humans and animals. Meanwhile, the blue group (B1–B4) addresses cost-effectiveness, time efficiency, sample consumption, and operational simplicity. The RGB12 model calculates scores for each of the three-color categories, which are then used to determine the overall average “whiteness” score, with 100 being the highest and a blended color, where the best outcome is white [33]. The evaluation of the proposed method according to the RGB12 algorithm is presented in Fig. S10. A score of 95.0% and 99.1% of the proposed method for plasma and urine determination, respectively, confirms the whiteness of the suggested voltammetric method and reflects the harmony of analytical and practical attributes.

### Blueness assessment

The blue applicability grade index (BAGI) is a novel metric for evaluating the practicality of analytical methods. Unlike conventional green metrics that emphasize environmental impact, BAGI focuses on ten essential factors influencing method applicability. These factors include the type of analysis, ability to detect multiple analytes simultaneously, availability of instrumentation, type of sample preparation, capacity for simultaneous sample preparation, number of samples processed per hour, choice of reagents and materials, need for preconcentration, level of automation, and the amount of sample required. An asteroid pictogram and corresponding score ranging from 25 to 100 in the center are generated, effectively highlighting the strengths and limitations of the method concerning its practicality and applicability. The pictogram’s color represents the compliance level, with dark blue signifying high compliance, blue for medium, light blue for low, and white for no compliance. A score of 25 implies poor applicability, while 100 shows excellent performance [34]. The asteroid pictogram of this work is shown in Fig. S11. with a score of 72.5 and 77.5 for plasma and urine determination, respectively. This reveals the good applicability and practicality of the Ni-MOF/CNTs/MCPE-based method.

## Conclusion

A sensitive electrochemical sensor was established by modifying a CPE with Ni-MOF/CNTs to determine sotagliflozin in various matrices. The synergistic integration of Ni-MOF and CNTs greatly enhances the electrocatalytic performance of the proposed electrode, increasing its electroactive surface area, reducing charge transfer resistance, and improving electron transfer kinetics and catalytic activity, resulting in a significant boost in the anodic current for sotagliflozin. Concerning sotagliflozin oxidation mechanism, it shows an irreversible oxidation involving the loss of two electrons and two protons with diffusion-controlled behavior. Under optimal conditions, the Ni-MOF/CNTs/CPE demonstrates a low detection limit (2.65 × 10^−10^ M) across wide linear ranges (8 × 10^−10^–6.0 × 10^−7^ M and 6.0 × 10^−7^–8.0 × 10^−5^ M). The fabricated electrode is reliable, sensitive, affordable, and user- and eco-friendly, making it a fruitful handheld tool in quality control labs and for therapeutic monitoring of sotagliflozin. The whiteness and blueness of the method are assessed using RGB12 and BAGI tools.

## Supplementary Information

Below is the link to the electronic supplementary material.

ESM 1 (DOCX 4.48 MB)

## Data Availability

Data is provided within the manuscript or supplementary information files.
