# Supplementary material for: Synergistically enhanced Ni-MOF/CNTs nanocomposite as an electrochemical platform for ultrasensitive determination of sotagliflozin in various matrices, with whiteness and blueness assessments
Source: Mikrochim Acta. 2025 May 26;192(6):374. doi: 10.1007/s00604-025-07235-5 (PMC12104113; doi:10.1007/s00604-025-07235-5)
Supplement: Supplementary file 1 — (DOCX 4.48 MB) [file 604_2025_7235_MOESM1_ESM.docx]

**Supplementary Material**

**Synergistically enhanced Ni-MOF/CNTs nanocomposite as an electrochemical platform for ultrasensitive determination of sotagliflozin in various matrices, with whiteness and blueness assessments**

Hend Z. Yamani^a^**^†^**^*^, Yasmine H. Hassan^a^**^†^**, Nancy Magdy^a^, Maha F. Abdel Ghany^a^, Mohammed M. Gomaa^b^

*^a^ Pharmaceutical Analytical Chemistry Department, Faculty of Pharmacy, Ain Shams University, Cairo 11566, Egypt*

*^b^ Solid State Physics Department, National Research Centre, Giza 12622, Egypt*

**^†^** The authors contributed equally to this work

*Corresponding author: Hend Z. Yamani

email: [hend.z.yamani@pharma.asu.edu.eg](mailto:hend.z.yamani@pharma.asu.edu.eg)

**
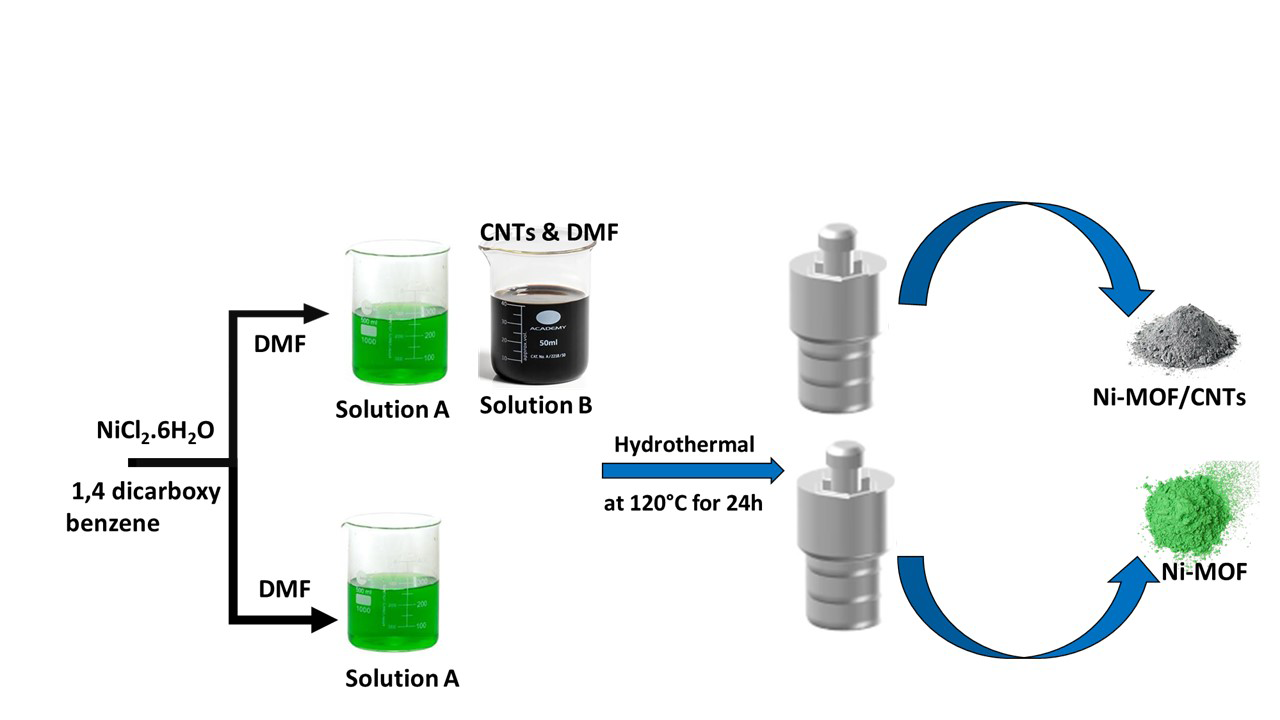
**

**Fig. S1.** Schematic diagram of the synthesis of Ni-MOF and Ni-MOF/CNTs nanocomposite


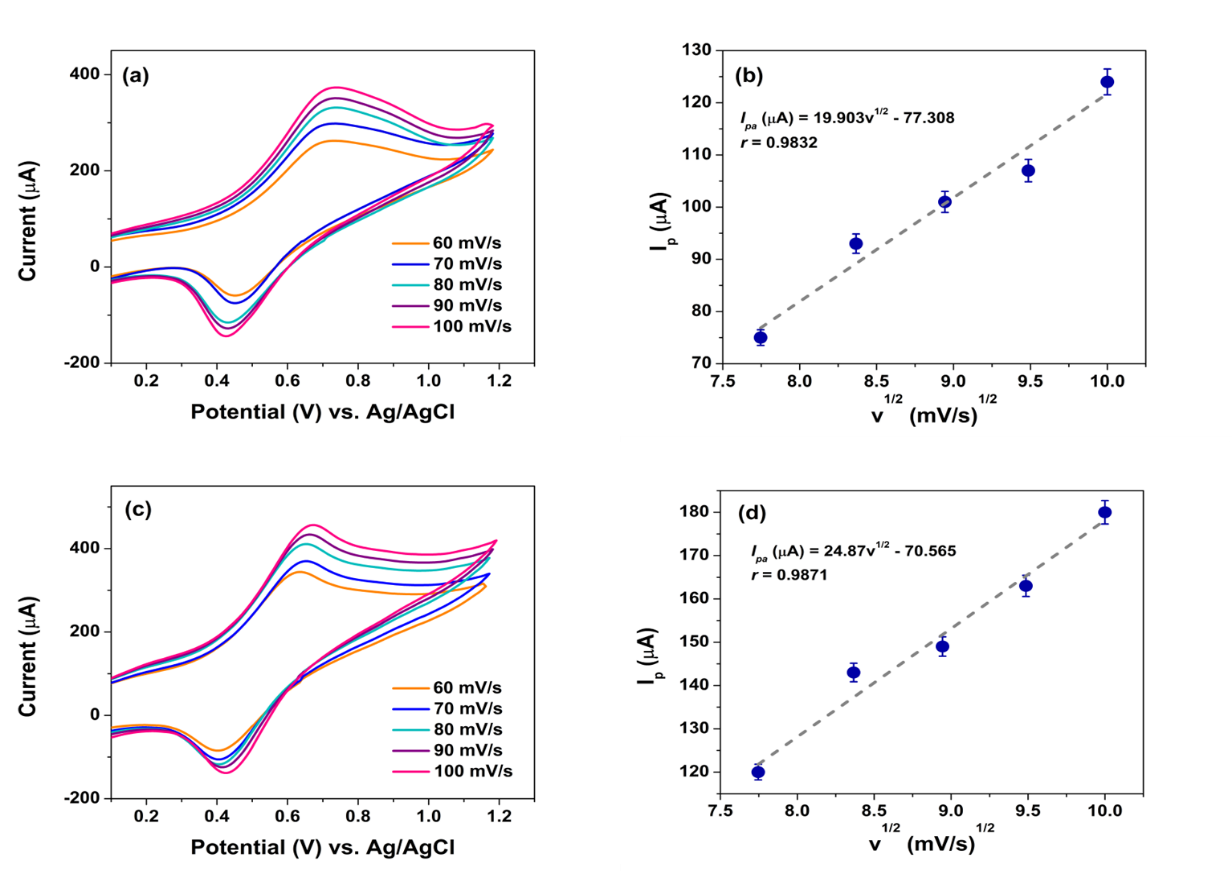


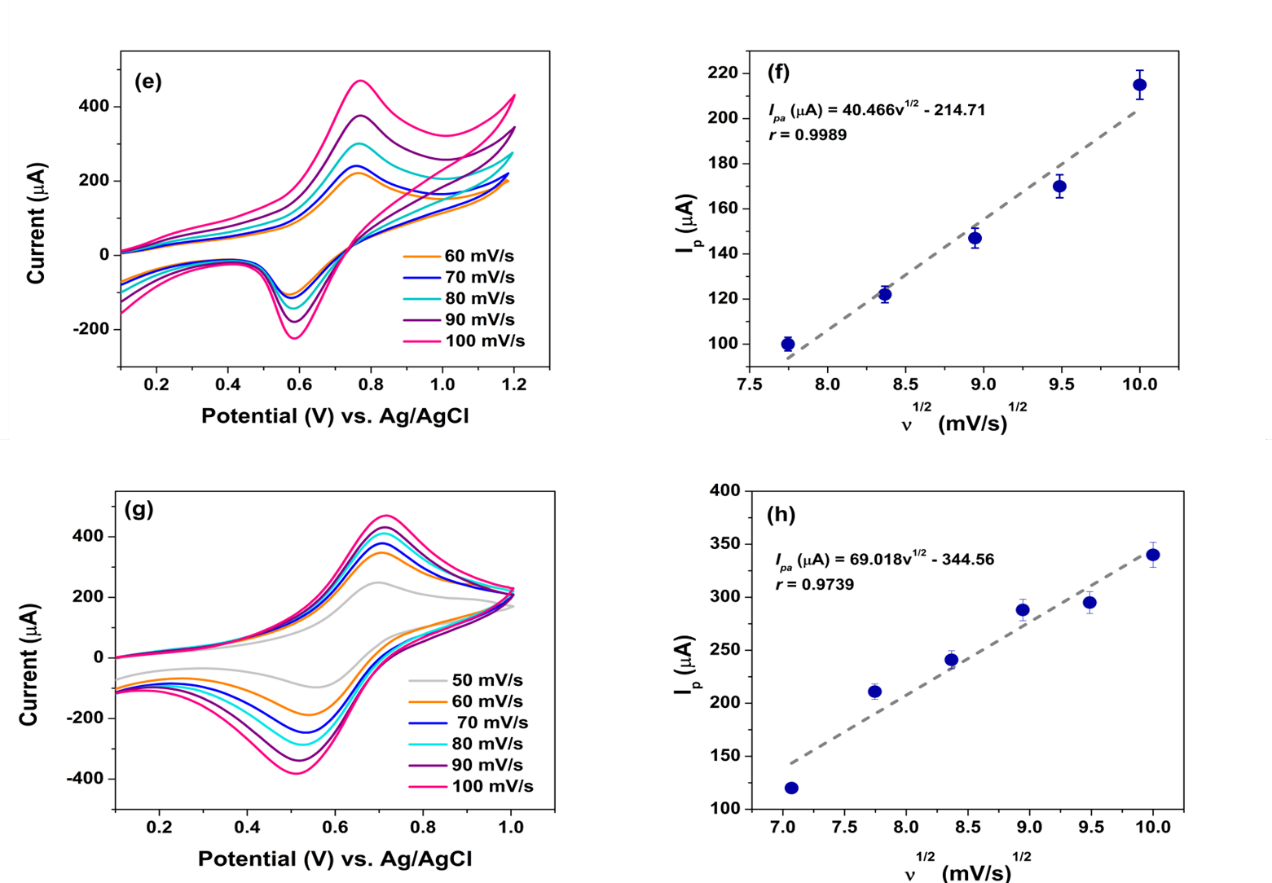


**Fig. S2.** CV responses of bare CPE, Ni-MOF/MCPE, CNTs/MCPE, and Ni-MOF/CNTs/MCPE in 1.0 mM K_3_Fe(CN)_6_^3−/4−^ in 0.1 M KCl at different scan rates


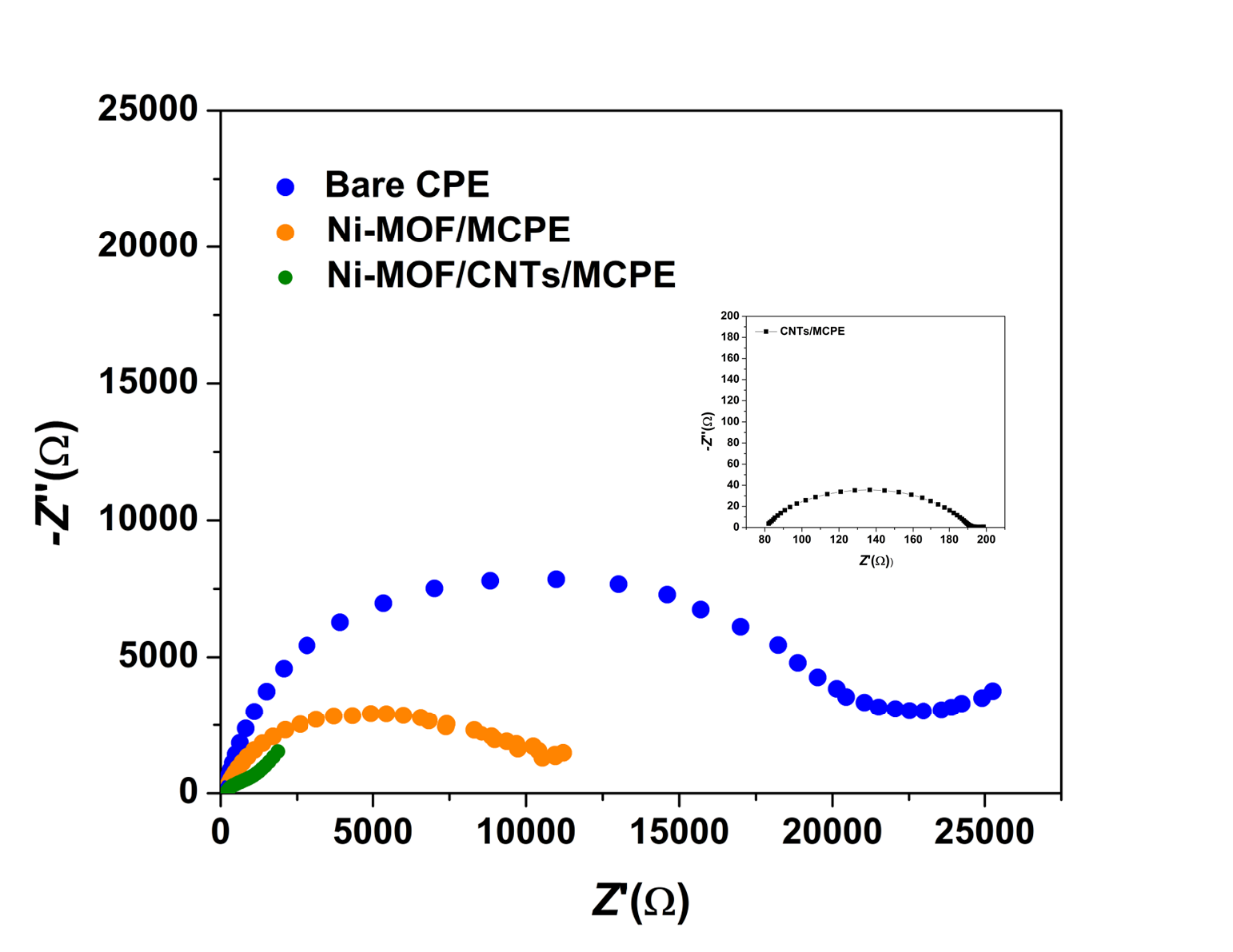


**Fig. S3.** Nyquist plots of bare CPE, Ni-MOF/MCPE, and Ni-MOF/CNTs/MCPE in 1.0 mM K_3_Fe(CN)_6_^3−/4−^ in 0.1 M KCl. The inset illustrates the Nyquist plot of CNTs/MCPE.


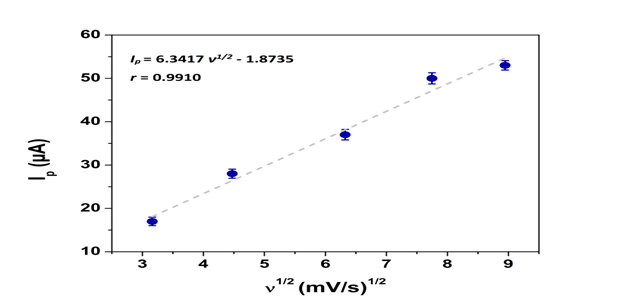


**Fig. S4.** The relation between anodic peak current and square root of scan rate


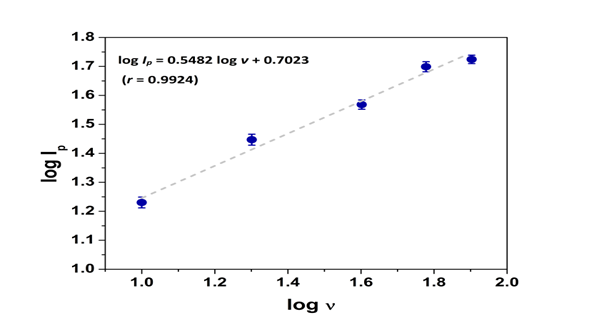


**Fig. S5**. The relation between log anodic peak current and log scan rate

**
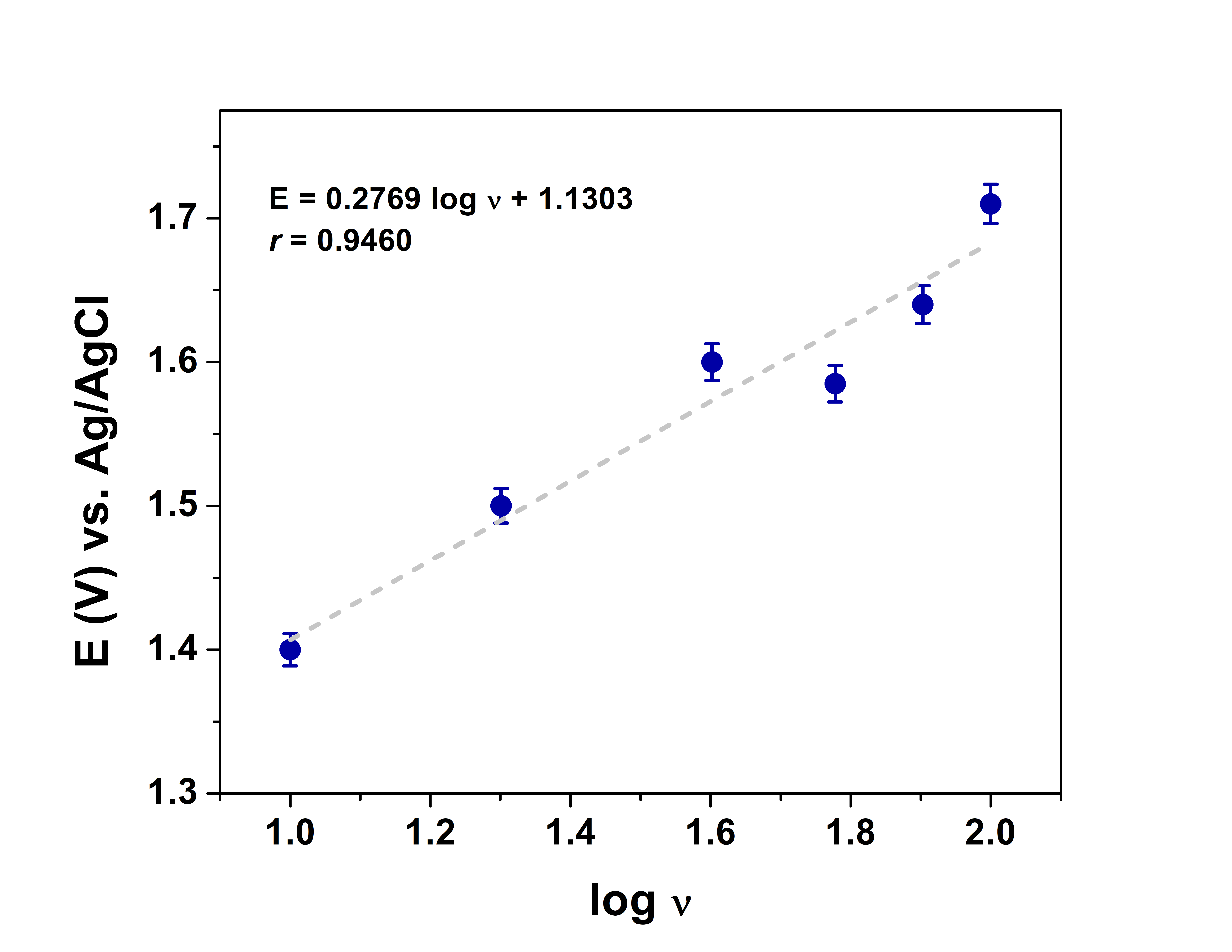
**

**Fig. S6.** The relation between peak potential and log scan rate


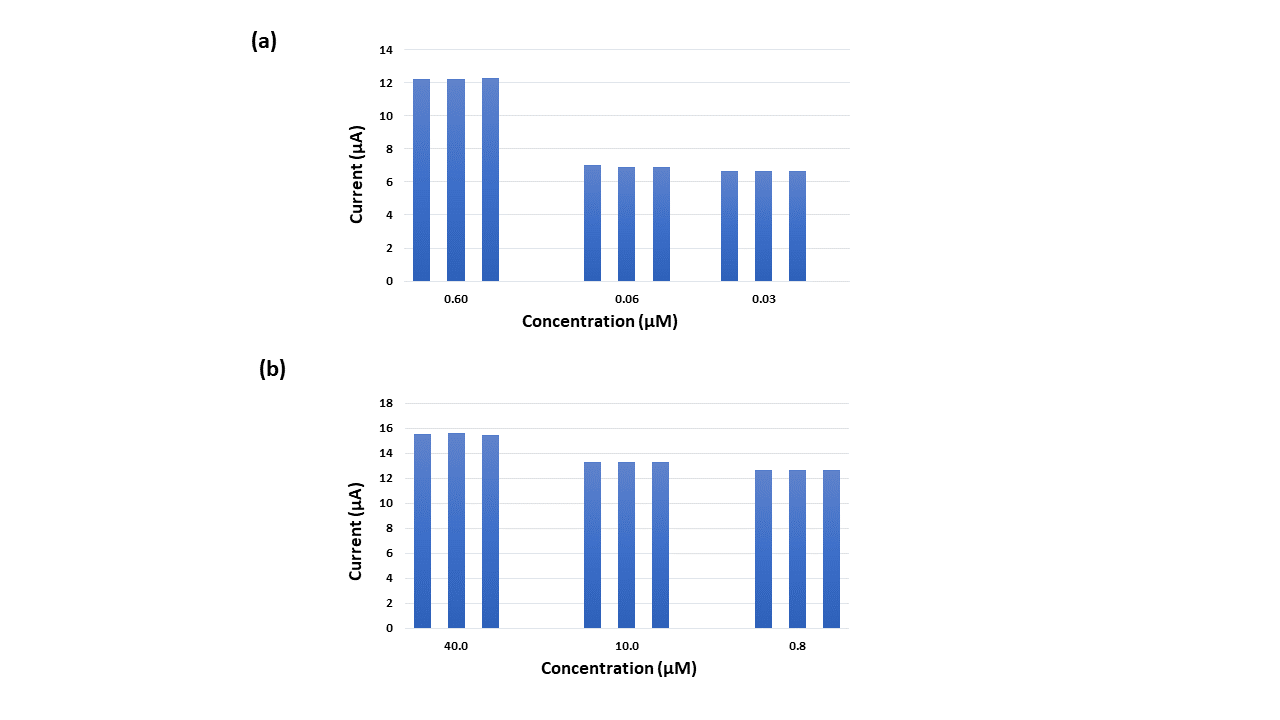


**Fig. S7.** Repeatability of DPV responses of Ni-MOF/CNTs/MCPE at concentration levels in the (a) lower linear range, and (b) higher linear range.

^
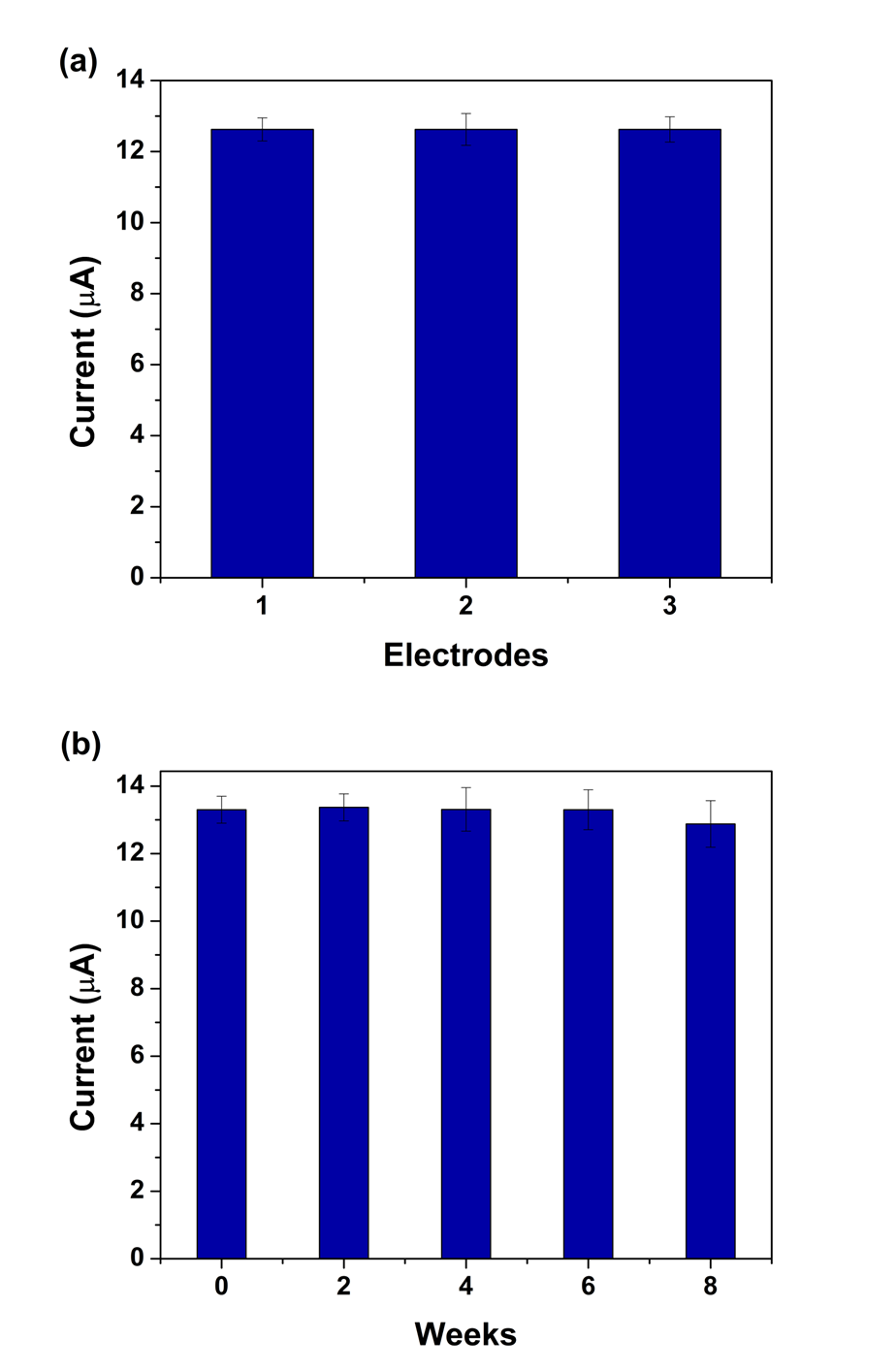
^

**Fig. S8.** (a) Reproducibility, and (b) stability of DPV responses Ni-MOF/CNTs/MCPE

**
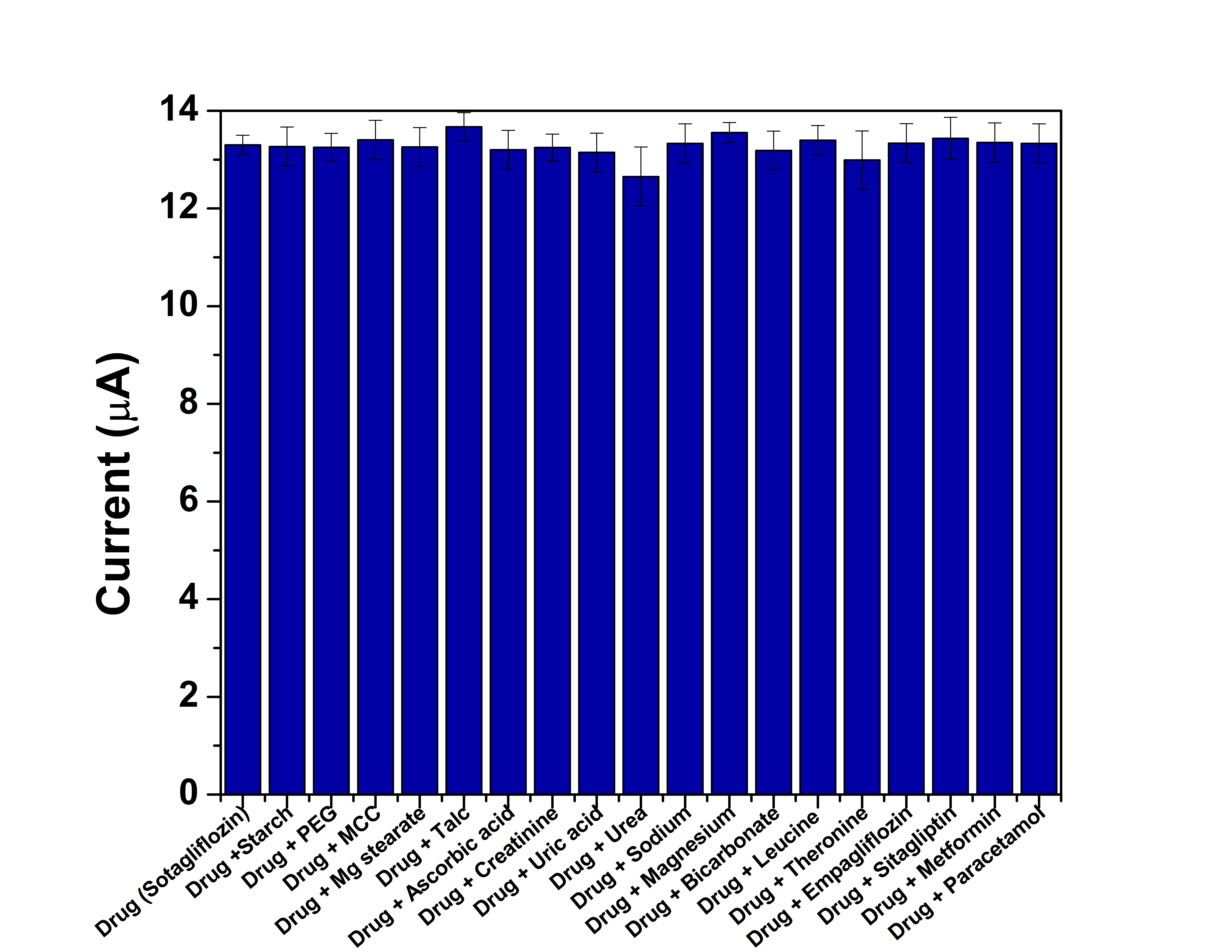
**

**Fig. S9.** Selectivity of Ni-MOF/CNTs/MCPE toward 1 x 10^-5^ M of sotagliflozin (in BRB of pH 10.0) in presence of different interferants


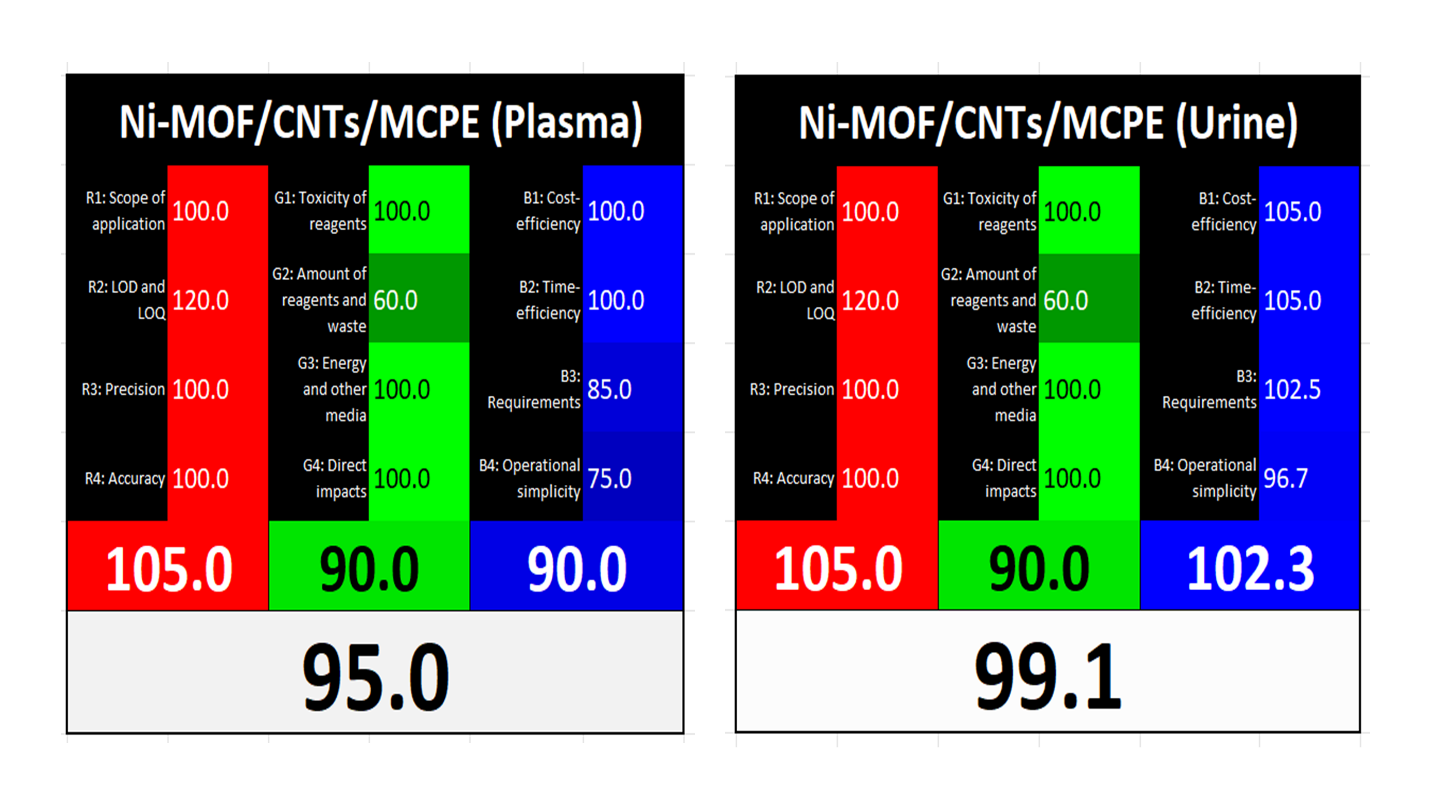
**Fig. S10.** Whiteness assessment using RGB12 algorithm for the proposed method


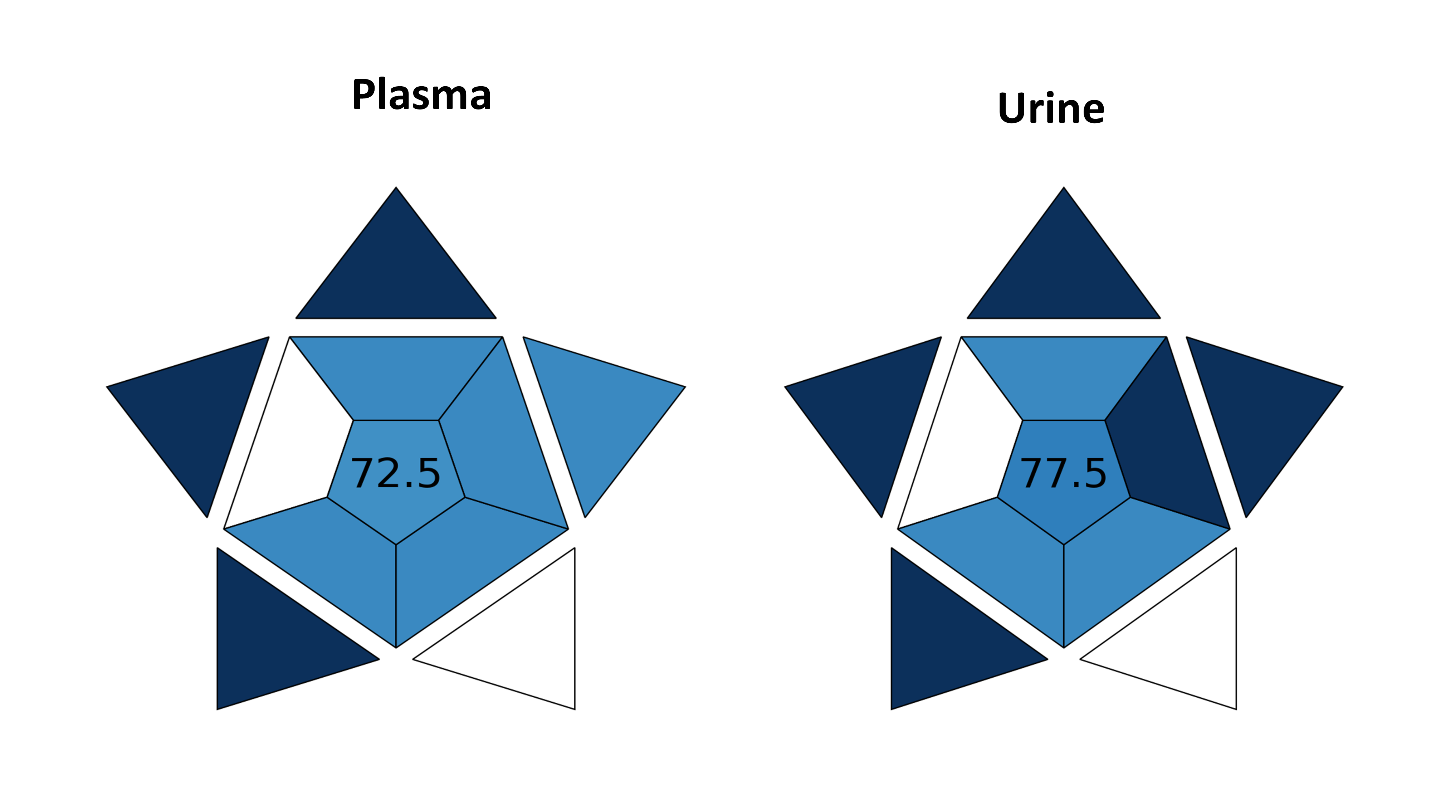
**Fig. S11.** Blueness assessment using BAGI index pictograms for the proposed method

**Table S1. Calculated *R_ct_*, *k^0^*, and *j_0_* for the bare CPE and MCPE**

| CNTs/MCPE | *R_ct_* (Ω) | *k^0^* (cm s^-1^) | *j_0_* (A cm^-^²) |
| --- | --- | --- | --- |
| Bare CPE | 20200 | 4.88 x 10^-4^ | 1.27 x 10^-6^ |
| Ni-MOF/MCPE | 10200 | 7.67 x 10^-4^ | 2.52 x 10^-6^ |
| CNTs/MCPE | 120 | 4.03 x 10^-2^ | 2.14 x 10^-4^ |
| Ni-MOF/CNTs/MCPE | 1440 | 1.95 x 10^-3^ | 1.78 x 10^-5^ |

**Table S2. Validation parameters of the proposed Ni-MOF/CNTs/MCPE based voltammetric method for determination of sotagliflozin**

| **Parameter** | **Lower liner range** | **Higher linear range** |
| --- | --- | --- |
| **Range** (M) | 8.0 x 10^-10^ – 6.0 x 10^-7^ | 6.0 x 10^-7^ – 8.0 x 10^-5^ |
| Correlation coefficient (*r*) | 0.9999 | 0.9998 |
| Slope | 10.140 | 0.07647 |
| Intercept | 6.3364 | 12.549 |
| Standard error of slope | 0.04389 | 0.00063 |
| Standard error of intercept | 0.01253 | 0.02433 |
| Confidence interval of slope^*^ | (10.030% , 10.256%) | (0.075%, 0.078%) |
| Confidence interval of intercept^*^ | (6.304% , 6.369%) | (12.487%, 12.612%) |
| **LOQ** (M) | 7.95 x 10^-10^ | --- |
| **LOD** (M) | 2.65 x 10^-10^ | --- |
| **Accuracy** (Mean ± %RSD)^**^ | 99.78 ± 1.438 | 98.53 ± 1.270 |
| **Precision** (%RSD)^**^ | | |
| Repeatability | 1.438 | 1.270 |
| Intermediate precision | 1.523 | 1.822 |

^*^Confidence intervals for the calibration plots were calculated at a 95% confidence level (lower, upper 95% CI).

^**^ Average of 3 replicates for each concentration

**Table S3. Selectivity evaluation of Ni-MOF/CNTs/MCPE towards sotagliflozin in the presence of different interferents**

| **Interferents** | **% Relative error in current^*^** |
| --- | --- |
| Starch | -0.24 |
| PEG | -0.38 |
| MCC | 0.77 |
| Magnesium stearate | -0.31 |
| Talc | 2.79 |
| Ascorbic acid | -0.73 |
| Creatinine | -0.39 |
| Uric acid | -1.16 |
| Urea | -4.87 |
| Na^+^ | 0.25 |
| Mg^2+^ | 1.91 |
| Bicarbonate | -0.85 |
| Leucine | 0.72 |
| Threonine | -2.32 |
| Empagliflozin | 0.27 |
| Sitagliptin | 1.02 |
| Metformin | 0.38 |
| Paracetamol | 0.24 |

**^*^** Average of three determinations
